# Well-defined coinage metal transfer agents for the synthesis of NHC-based nickel, rhodium and palladium macrocycles†
†Electronic supplementary information (ESI) available: ^1^H and ^13^C{^1^H} NMR, and ESI-MS spectra of new complexes. Selected ^1^H NMR data for transmetallation reactions of **2** and **4**. CCDC 1470494–1470497. For ESI and crystallographic data in CIF or other electronic format see DOI: 10.1039/c6dt01263a



**DOI:** 10.1039/c6dt01263a

**Published:** 2016-05-09

**Authors:** Rhiann E. Andrew, Caroline M. Storey, Adrian B. Chaplin

**Affiliations:** a Department of Chemistry , University of Warwick , Gibbet Hill Road , Coventry CV4 7AL , UK . Email: a.b.chaplin@warwick.ac.uk

## Abstract

With a view to use as carbene transfer agents, well-defined silver(i) and copper(i) complexes of a macrocyclic NHC-based pincer ligand, bearing a central lutidine donor and a dodecamethylene spacer [CNC–(CH_2_)_12_, **1**], have been prepared. Although the silver adduct is characterised by X-ray diffraction as a dinuclear species *anti*-[Ag(μ-**1**)]_2_^2+^, variable temperature measurements indicate dynamic structural interchange in solution involving fragmentation into mononuclear [Ag(**1**)]^+^ on the NMR time scale. In contrast, a mononuclear structure is evident in both solution and the solid-state for the analogous copper adduct partnered with the weakly coordinating [BAr^F^_4_]^–^ counter anion. A related copper derivative, bearing instead the more coordinating cuprous bromide dianion [Cu_2_Br_4_]^2–^, is notable for the adoption of an interesting tetranuclear assembly in the solid-state, featuring two cuprophilic interactions and two bridging NHC donors, but is not retained on dissolution. Coinage metal precursors [M(**1**)]_*n*_[BAr^F^_4_]_*n*_ (M = Ag, *n* = 2; M = Cu, *n* = 1) both act as carbene transfer agents to afford palladium, rhodium and nickel complexes of **1** and the effectiveness of these precursors has been evaluated under equivalent reaction conditions.

## Abstract 2

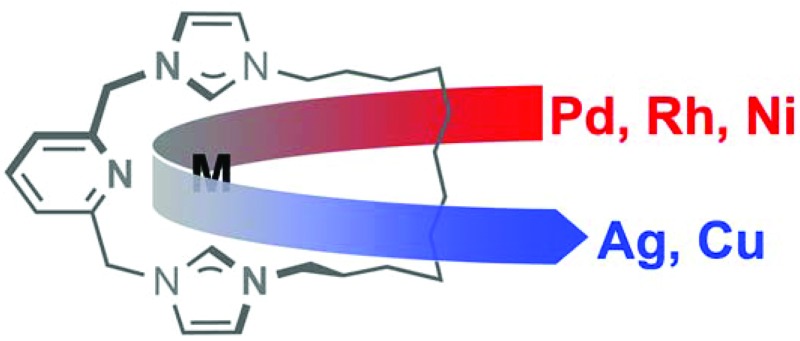
Silver(i) and copper(i) complexes of a macrocyclic NHC-based pincer ligand, bearing a central lutidine donor and a dodecamethylene spacer, have been prepared and evaluated under equivalent conditions in transmetallation reactions.

## Introduction

**Scheme 1:**
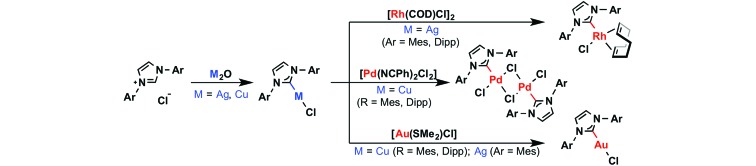
Selected synthesis and reactions of Cu(i) and Ag(i)–NHC complexes.^4,5^

The chemistry of N-heterocyclic carbene (NHC) complexes of the transition elements is rich, varied and at the forefront of contemporary organometallic chemistry and catalysis.^1^ While the formation of these adducts *via* direct coordination of the singlet carbene, isolated or generated *in situ* through deprotonation of the corresponding azolium salt, is conceptually the simplest method, the high chemical reactivity of these free carbene intermediates or incompatibility of the reaction conditions with acidic functionalities have necessitated the development of alternative approaches involving isolable ‘protected’ carbenes and transmetallation procedures. In this context, Ag(i)–NHC complexes have proved to be effective carbene transfer agents for a wide variety of late-transition elements (Scheme 1).^2–4^ These reagents are conveniently formed through reaction of the respective pro-ligand NHC·HX with Ag_2_O, resulting in mono- or bis-ligated complexes [Ag(NHC)X] or [Ag(NHC)_2_]X depending on the nature of the anion (X).^2,3^ Although comparatively less common, Cu(i)–NHC complexes have also been employed as carbene transfer agents (Scheme 1).^2,5^ Such copper reagents can be prepared from reactions between Cu_2_O and NHC·HX, but are generally prepared *via* free carbene intermediates.^2^ Interestingly, reflecting the relative M(i)–NHC bond strengths of the coinage metals (Au > Cu > Ag),^6^ transmetallation of both silver and copper complexes can be used to prepare gold derivatives as a result of the more robust nature of Au(i)–NHC bonds.^2–5^

**Scheme 2:**
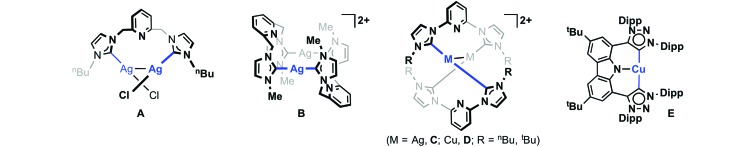
Examples of isolated Ag(i) and Cu(i) complexes of CNC pincer ligands.^12–17^

The application of carbene transfer methodology is not limited to monodentate examples and the use of silver-based transmetallation protocols is also prevalent in the coordination chemistry of *mer*-tridentate “pincer” ligands bearing terminal NHC donors.^7,8^ While the corresponding silver transfer agents are often generated *in situ*, well defined and characteristically bimetallic Ag(i)–CEC (E = C, N) complexes such as **A–C** have been isolated and crystallographically characterised (Scheme 2).^9–15^ Copper adducts of CEC (E = C, N) ligands have also been prepared (*e.g.* **D** and **E**),^10,16–18^ however, their application as transfer agents has yet to be realised to our knowledge.

As part of our work involving the organometallic chemistry of macrocyclic CNC pincers,^19^ we now report the synthesis and characterisation of well-defined Ag(i) and Cu(i) adducts of a lutidine-based pincer ligand bearing a dodecamethylene spacer [CNC–(CH_2_)_12_, **1**]. The use of these coinage metal species as transfer agents is then detailed for the synthesis of rhodium, palladium, and nickel complexes of **1**.

## Results and discussion

**Scheme 3:**
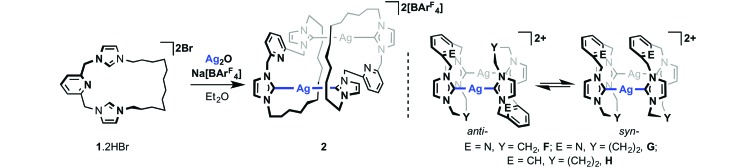
Preparation of **2** and related literature precedents.^11^

Our preceding work exploring the coordination chemistry of **1** employed *in situ* generation of silver transfer agents from the reaction between **1**·2HBr and Ag_2_O in CH_2_Cl_2_ solution.^19^ As a convenient means of incorporating a weakly coordinating [BAr^F^_4_]^–^ anion^20^ and conferring solution solubility, these reactions were carried out in the presence of Na[BAr^F^_4_] (Ar^F^ = 3,5-C_6_H_3_(CF_3_)_2_) as halogen ion abstractor. Using a slightly adapted protocol to facilitate isolation, well-defined silver derivative **2** was prepared *via* reaction in diethyl ether (Scheme 3). Filtration to remove insoluble silver and sodium bromide salts, and subsequent recrystallization from CHCl_3_/pentane, afforded analytically pure **2** as a white powder in reproducibly high isolated yields of *ca.* 80%.

**Fig. 1:**
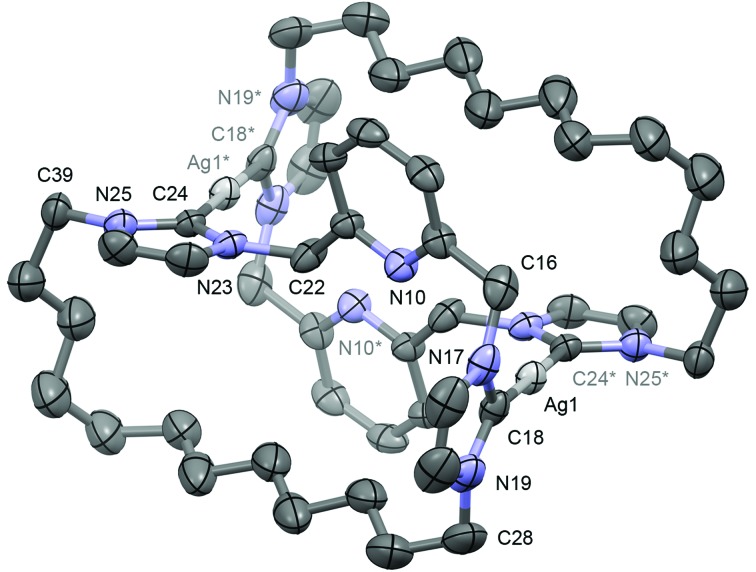
Solid-state structure of **2**. Thermal ellipsoids drawn at the 50% probability level; hydrogen atoms, minor disordered components and anions omitted for clarity. The starred atoms are generated by the symmetry operation 2 – *x*, 1 – *y*, 1 – *z*. Selected bond lengths (Å) and angles (°): Ag1–C18, 2.077(3); Ag1–C24*, 2.080(3); Ag1···Ag1*, 7.2732(6); C24–Ag1–C18*, 178.99(13); N25–C24–C18*–N19*, 100.2(4).

Single crystals grown from diethyl ether/pentane and analysed by X-ray crystallography enabled structural elucidation of **2** in the solid-state as a dinuclear complex *anti*-[Ag(μ-**1**)]_2_[BAr^F^_4_]_2_ (Fig. 1) – as for closely related precedents **B**, **F** and **G**.^11,13^ Interestingly, while the principle geometric metrics about silver in **2** are directly comparable to the aforementioned precedents (*e.g.* Ag–NHC = 2.088(4), 2.093(4) Å, **B**; 2.077(3), 2.080(3) Å, **2**; NHC–Ag–NHC = 176.78(9)°, **B**; 178.99(13)°, **2**), the ligand topology is significantly altered. At the heart of the structural difference is the adoption of near orthogonal NHC–Ag–NHC geometries in **2** [N25–C24–C18*–N19* = 100.2(4)°], in contrast to coplanar NHC–Ag–NHC arrangements observed in **B**, **F** and **G**. This change in geometry is presumably necessary to accommodate the long aliphatic chain and as a consequence results in a very large Ag···Ag* separation [7.2732(6) *cf.* 3.7171(5), **B**; 3.538(2), **F**; 4.6636(7) Å, **G**], precluding the adoption of any argentophilic interaction as seen in **A** and **C**.^12,14,15^.

^1^H and ^13^C NMR data recorded in CD_2_Cl_2_ confirm the expected 1 : 1 ligand to anion ratio and reveal time averaged *C*_2v_ symmetry for **2** at 298 K (500 MHz). Such high symmetry is inconsistent with retention of the solid-state structure and instead implies highly fluxional behaviour in solution, involving fragmentation into [Ag(**1**)]^+^ (*cf.* structure of **4** *vide infra*). Such an assertion is supported by ESI-MS, where only a singly charged species (*i.e.* integer mass spacing) was evident in the mass spectrum ([Ag(**1**)]^+^, 512.1927; calc. 512.1938 *m*/*z*). Not surprisingly the carbene signals of **2** were not observed by ^13^C{^1^H} NMR spectroscopy at 298 K, although a chemical shift of *ca. δ* 181 ppm can be inferred from 2D heteronuclear correlation experiments in line with expected values for Ag(i)–NHC complexes.^2,3b,21^ Progressive cooling to 250 K lead to decoalescence of the (broadened) ^1^H signals of **2** observed in CD_2_Cl_2_ at 298 K and establishment of an equilibrium mixture comprised of three major compounds, one of *C*_2v_ symmetry and two of apparent *C*_s_ symmetry (1 : 0.5 : 1). These species are tentatively assigned as [Ag(**1**)]^+^, *syn*-[Ag(μ-**1**)]_2_^2+^ and *anti*-[Ag(μ-**1**)]_2_^2+^, respectively, on the basis of related behaviour observed for **F** and **G** involving rapid equilibration between *syn*- and *anti*-isomers in solution (Scheme 3); dynamics that necessitate Ag–NHC bond cleavage and invoke coordination of the central lutidine donor through comparison to the less fluxional *m*-xylylene bridged analogue **H**.^11^ In the case of **2**, the NMR data suggests that incorporation of the long dodecamethylene spacer destabilises the dinuclear structures relative to *entropically* favored fragmentation into [Ag(**1**)]^+^ at ambient temperature.^22^ Further cooling to 200 K resulted in a shift in the equilibrium toward the species assigned to *anti*-[Ag(μ-**1**)]_2_^2+^ (*ca.* 60%) consistent with the pseudo *C*_i_ symmetric structure observed in the solid-state being *enthalpically* favoured in solution. In the context of **2** being used as a carbene transfer agent, these NMR data ultimately demonstrate facile Ag–NHC bond cleavage under conditions relevant to synthesis of other transition metal adducts of **1** *via* transmetallation.

**Scheme 4:**
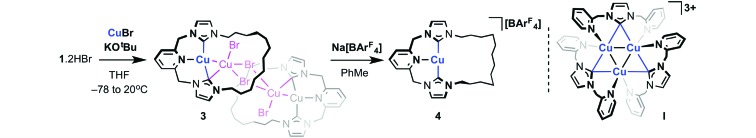
Preparation of **3**, **4** and a related literature precedent.^25^

The synthesis of copper adducts of **1** was targeted by low temperature deprotonation of **1**·2HBr in THF in the presence of excess copper bromide. In this manner [Cu(**1**)]_2_[Cu_2_Br_4_] **3** was formed and subsequently isolated in 76% yield (Scheme 4). Further treatment of **3** with Na[BAr^F^_4_] in toluene resulted in incorporation of the weakly coordinating [BAr^F^_4_]^–^ anion in place of [Cu_2_Br_4_]^2–^ to afford **4** in 59% isolated yield.^20,23^ In CD_2_Cl_2_ solution, the ^1^H and ^13^C NMR characteristics of both **3** and **4** point towards simple mononuclear complexes of **1**, with sharp resonances and apparent *C*_2v_ symmetry in the respective spectra at 298 K (400 MHz). Moreover, only minor differences in chemical shift are found for the equivalent ^1^H (<0.15 ppm) and ^13^C (<2 ppm) signals of **3/4**, and presumably attributed to greater ion pairing in **3**. Of most relevance to the coordination of **1**, the carbenic centres were readily identified from ^13^C{^1^H} NMR spectra by their characteristically high frequency chemical shifts (*δ* 178.7, **3**; *δ* 180.3, **4**).^2^ Strong parent cation signals are observed by ESI-MS with correct isotope patterns and integer mass spacing (468.2185, **3**; 468.2186, **4**; calc. 468.2183 *m*/*z*), further supporting the presence of discrete [Cu(**1**)]^+^ in solution, irrespective of the counter anion.

**Fig. 2:**
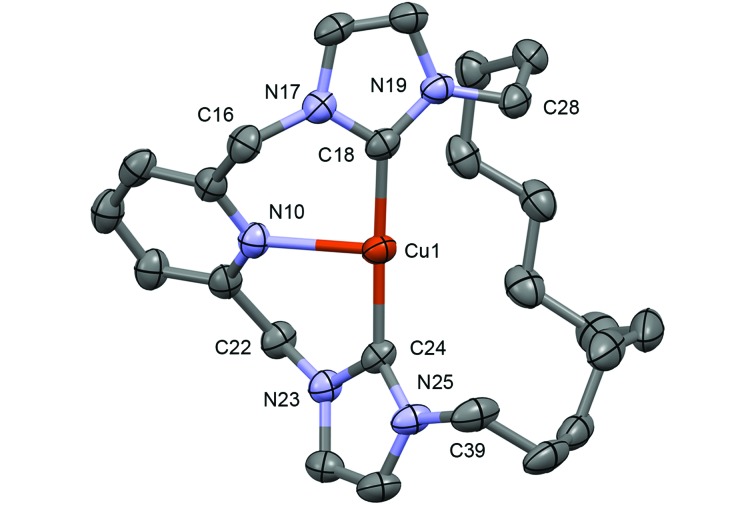
Solid-state structure of **4**. Thermal ellipsoids drawn at the 50% probability level; only one of the unique cations shown (*Z*′ = 2) and hydrogen atoms omitted for clarity. Selected bond lengths (Å) and angles (°): Cu1–N10, 2.233(3); Cu1–C18, 1.905(3); Cu1–C24, 1.906(3); C18–Cu1–C24, 176.37(13).

**Fig. 3:**
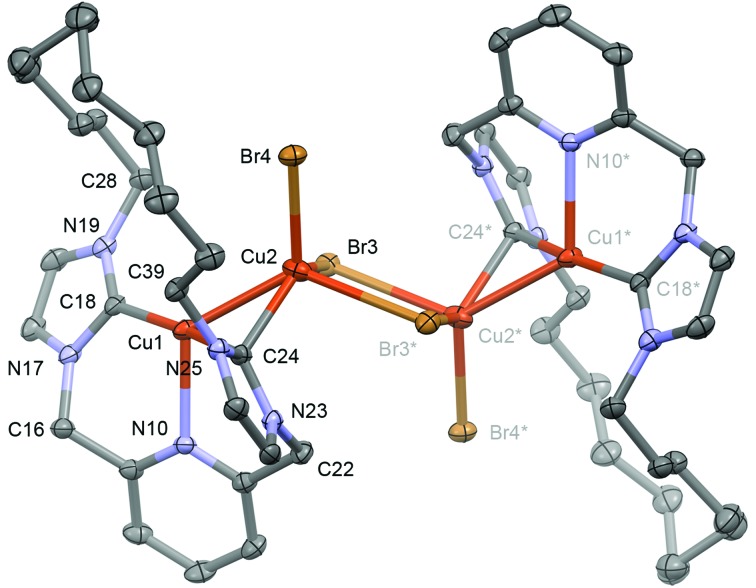
Solid-state structure of **3**. Thermal ellipsoids drawn at the 50% probability level; hydrogen atoms omitted for clarity. The starred atoms are generated by the symmetry operation 1 – *x*, 1 – *y*, 1 – *z*. Selected bond lengths (Å) and angles (°): Cu1–Cu2, 2.5209(5); Cu1–N10, 2.246(2); Cu1–C18, 1.925(3); Cu1–C24, 1.980(3); Cu2–Br3, 2.4583(4); Cu2–Br3*, 2.7728(5); Cu2–Br4, 2.4149(4); Cu2–C24, 2.070(3); Cu2–Br3–Cu2*, 83.794(15); Br3–Cu2–Br3*, 96.206(15); Cu1–Cu2–C24, 49.92(7); C18–Cu1–C24, 167.92(11).

Despite similar solution characteristics, in the solid-state the nature of the counter anion impacts significantly on the coordination geometries of **3** and **4** (Fig. 2 and 3). Two independent, but well-separated cation/anion pairs are observed in the solid-state structure of **4**. The cationic fragments are structurally similar, displaying near ideal T-shaped coordination geometries with the flexible dodecamethylene spacer notably skewed to one side. Focusing on the metrics associated with the independent cation shown in Fig. 2,^24^ the complex displays an approximately linear NHC–Cu–NHC angle (176.37(13)°), equivalent Cu–NHC bond lengths within error (1.905(3)/1.906(3) Å), and a Cu–N bond length of 2.233(3) Å. These parameters are in good agreement with those reported for **E**, although the anionic nature of the component CNC pincer ligand leads to a shorter Cu–N bond length than that in **4** (2.017(2) Å). In the case of **3**, an anion bridged dimer/tetranuclear formulation is instead observed, *viz* [Cu(**1**)]_2_{μ-[Cu_2_Br_4_]}, featuring two formally bridging NHC donors (Cu1–C24, 1.980(3); Cu2–C24, 2.070(3) Å) and two cuprophilic interactions (Cu–Cu, 2.5209(5) Å). The bonding interaction with the anion results in a significant distortion of the coordination geometry observed in **4**, with the NHC–Cu–NHC angle deviating from linearity (167.92(11)°) alongside elongation of the non-bridged Cu–NHC (1.925(3) *cf.* 1.905(3)/1.906(3) Å) and Cu–N (Cu–N = 2.246(2) *cf.* 2.233(3)) bonds. The bridging μ^2^-NHC coordination mode is an unusual feature of **3**, but has striking precedence in trinuclear copper clusters, such as **I**, that contain three ligands in this coordination mode (Cu–NHC *ca.* 2.026(5)–2.044(5) Å for **I**).^25,26^ In these trinuclear clusters, the bridging coordination mode is retained in solution and characterised by *δ*_13C_ 167–169 for imidazolyline based variants; values to significantly lower frequency than found for **3** (*δ*_13C_ 178.7).^25,26^

With well-defined coinage metal complexes **2** and **4** in hand, we turned to evaluation of their capacity to act as transfer agents of macrocyclic **1** in CH_2_Cl_2_. As convenient benchmarks we targeted preparation of known and soluble palladium and rhodium adducts of **1**, [Pd(**1**)Cl][BAr^F^_4_] **5** and [Rh(**1**)(CO)][BAr^F^_4_] **6**, through reactions with [Pd(NCMe)_2_Cl_2_] and [Rh(CO)_2_Cl]_2_, respectively (Table 1).^19b,c^ Following these reactions *in situ* by ^1^H NMR spectroscopy in CD_2_Cl_2_, using the [BAr^F^_4_]^–^ resonances as a convenient internal standard, revealed rapid and high yielding transmetallation reactions of **2** in both cases (>70% yield). Consistent with these high yields, **5** and **6** have previously been isolated in 58% and 52% yield, respectively, through *in situ* generation of the silver transfer agent.^19b,c^ In the case of copper, although complete consumption of **4** was apparent within 30 min in both cases, a large difference in selectivity is apparent: rhodium-based **6** was formed with an excellent yield of 98% (and subsequently isolated in 82% yield), while palladium-based **5** was formed with a significantly inferior yield of 23%.

**Table 1:** Transmetallation reactions of **2** and **4** ^*a*^

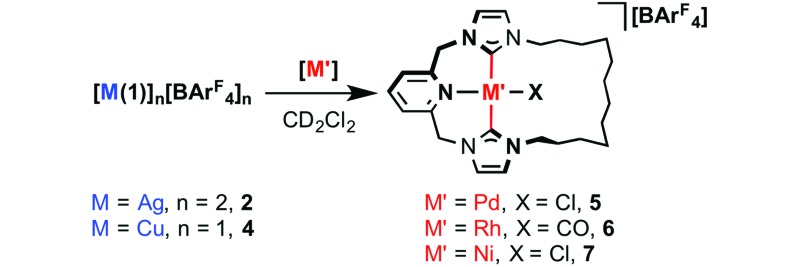					
[M′]	M	*t* ^*b*^/h	*T*/°C	Product	Yield^*c*^
[Pd(NCMe)_2_Cl_2_]	Ag	0.5	20	**5**	73%
[Pd(NCMe)_2_Cl_2_]	Cu	0.5	20	**5**	23%
[Rh(CO)_2_Cl]_2_	Ag	0.5	20	**6**	72%
[Rh(CO)_2_Cl]_2_	Cu	0.5	20	**6**	98%
[NiCl_2_(glyme)]	Ag	20^*d*^	20	**7**	22%
[NiCl_2_(glyme)]	Ag	20	40	**7**	76%
[NiCl_2_(glyme)]	Cu	20^*d*^	20	**7**	86%
[NiCl_2_(glyme)]	Cu	5	40	**7**	90%

^*a*^Reactions carried out in J. Young's NMR tubes, which were periodically placed in a ultrasound bath during the course of the reaction.

^*b*^Complete conversion unless otherwise noted.

^*c*^Determined by integration of ^1^H NMR data.

^*d*^Incomplete reaction.

Seeking to expand the scope of this transmetallation methodology, we targeted the preparation of nickel derivative **7** – the lighter and Earth abundant group 10 congener of **5**. Using the aforementioned methodology in combination with [NiCl_2_(glyme)], resulted in slow dissolution of the largely insoluble nickel(ii) precursor, and gratifying (albeit gradual) formation of **7** at room temperature over 20 hours. The reaction with copper-based **4** notably proceeded *ca.* 4 times faster, suggesting the explanation for this behaviour is more complex than low solubility of [NiCl_2_(glyme)] alone. Repeating under more forcing conditions (40 °C) resulted in complete consumption of **2** (20 h) and **4** (5 h) and formation of **7** in 76% and 90% yield, respectively. The new air and moisture stable complex **7** was subsequently isolated from these reactions in *ca.* 30% yield following purification on alumina and fully characterised. Alternatively, **7** can also be prepared in similar yield using *in situ* generation of **2** from **1**·2HBr and Ag_2_O (29% isolated yield).

**Fig. 4:**
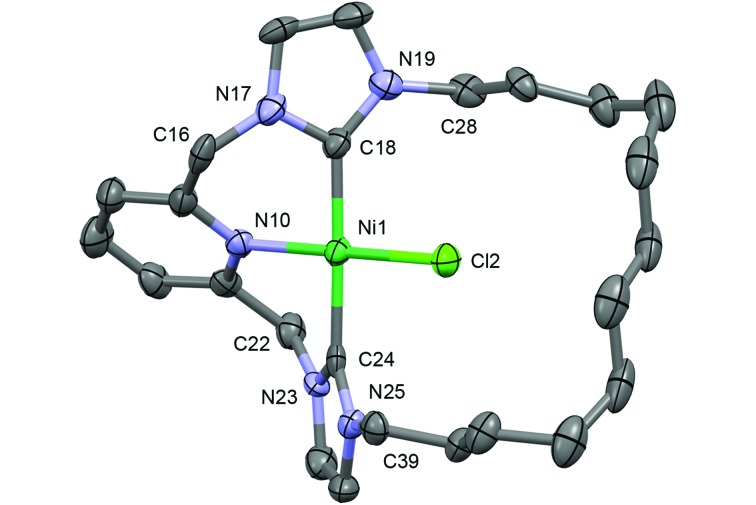
Solid-state structure of **7**. Thermal ellipsoids drawn at the 50% probability level; hydrogen atoms and anion omitted for clarity. Selected bond lengths (Å) and angles (°): Ni1–Cl2, 2.145(2); Ni1–N10, 1.928(5); Ni1–C18, 1.898(6); Ni1–C24, 1.916(6); N10–Ni1–Cl2, 179.06(15); C18–Ni1–C24, 176.6(3).

In the solid-state, **7** shows the expected contraction of metal–ligand bond lengths in comparison to **5** (Ni–Cl, 2.145(2); Ni–N, 1.928(5); Ni–C, 1.898(6), 1.916(6) Å; Pd–Cl, 2.287(4); Pd–N, 2.077(10); Pd–C, 2.036(12), 2.056(13) Å), but is otherwise isostructural with the palladium-based analogue (Fig. 4).^19*c*^ Most notably, the chloride ancillary ligand is easily accommodated within the macrocyclic ring, which is orientated to maintain pseudo *C*_2_ symmetry (*cf.* twisting observed in **4**), with an essentially linear N–Ni–Cl bond angle (179.06(15) *cf.* 176.2(3)° for the N–Pd–Cl angle in **5**). In solution the solid-state structure is fully retained as indicated by the observation of diastereotopic methylene bridge (pyCH_2_) and *N*-methylene (*N*-CH_2_CH_2_) resonances at *δ* 5.14/6.30 (^2^ *J*_HH_ = 15.0 Hz) and *δ* 3.73/4.73, respectively. A single carbenic carbon signal is observed at *δ* 162.0 (*cf. δ* 164.5, **5**).

## Summary

Well-defined silver(i) and copper(i) complexes of a macrocyclic NHC-based pincer ligand, bearing a central lutidine donor and a dodecamethylene spacer [CNC–(CH_2_)_12_, **1**], have been prepared. In the solid-state silver adduct **2** is characterised by X-ray diffraction as a dinuclear species *anti*-[Ag(μ-**1**)]_2_^2+^, with the two metal centres held distant from each other (Ag···Ag, >7 Å) as a consequence of the conformation of the bridging macrocyclic ligand. However, variable temperature ^1^H NMR spectroscopy indicates dynamic structural interchange in solution involving fragmentation into mononuclear [Ag(**1**)]^+^. In contrast, a mononuclear structure is evident in both solution and the solid-state for the analogous copper adduct **4** partnered with the weakly coordinating [BAr^F^_4_]^–^ counter anion. A related copper derivative, bearing instead the more coordinating cuprous bromide dianion [Cu_2_Br_4_]^2–^, is notable for the adoption of an interesting tetranuclear assembly in the solid-state, featuring two cuprophilic interactions (Cu–Cu, 2.5209(5) Å) and two bridging NHC donors, but is not retained in solution. Coinage metal complexes [M(**1**)]_*n*_[BAr^F^_4_]_*n*_ (M = Ag, *n* = 2, **2**; M = Cu, *n* = 1, **4**) both act as carbene transfer agents to afford palladium (**5**), rhodium (**6**) and nickel complexes (**7**) of **1**. Although not extensive, these reactions suggest that while silver-based transfer agents are more reliable, copper-based alternatives can result in significantly faster and higher yielding transmetallation reactions.

## Experimental

### General experimental methods

Manipulations were performed under an inert atmosphere, using Schlenk and glove box techniques unless otherwise stated. Glassware was oven dried and flamed under vacuum prior to use. Anhydrous solvents (<0.005% H_2_O) were purchased from ACROS or Aldrich and used as supplied: Et_2_O, CHCl_3_, pentane, CH_2_Cl_2_, THF and toluene. CD_2_Cl_2_ was dried over molecular sieves (4 Å) and stored under an atmosphere of argon. Na[BAr^F^_4_],^27^ [Rh(CO)_2_Cl]_2_,^28^ and **1**·2HBr^19*c*^ were synthesised using literature procedures. All other reagents are commercial products and were used as received. NMR spectra were recorded on Bruker HD-300, DPX-400, AV-400, DRX-500 and AVIII-500 HD spectrometers at 298 K unless otherwise stated. Chemical shirts are quoted in ppm and coupling constants in Hz. ESI-MS were recorded on a Bruker MaXis mass spectrometer. Microanalyses were performed at the London Metropolitan University by Stephen Boyer.

### Synthesis of **2**

A mixture of **1**·2HBr (100 mg, 0.176 mmol), Ag_2_O (42 mg, 0.181 mmol) and Na[BAr^F^_4_] (170 mg, 0.192 mmol) was suspended in Et_2_O (5 mL) and stirred under argon in the absence of light for 48 h. The resulting grey suspension was allowed to settle and the solution filtered through a celite plug (pipette, 3 cm) under a flow of nitrogen. The colourless filtrate was concentrated to afford the crude product as white foam, which was subsequently dissolved in hot chloroform and filtered through a second celite plug (pipette, 3 cm). The analytically pure product was obtained on addition of excess pentane, washed with pentane and dried. Yield: 200 mg (82%, white powder). Crystals suitable for X-ray diffraction were grown from ether/pentane at 20 °C.

**^1^H NMR** (400 MHz, CD_2_Cl_2_) *δ* 7.82 (br, 1H, py), 7.70–7.75 (m, 8 H, Ar^F^), 7.56 (br, 4H, Ar^F^), 7.42 (br, 2H, py), 7.17 (br, 2H, imid), 7.05 (s, 2H, imid), 5.25 (br, 4H, pyCH_2_), 4.06 (app. t, *J* = 7, 4H, NCH_2_), 1.79 (app. pent., *J* = 7, 4H, CH_2_) 1.10–1.40 (m, 16 H, CH_2_). ^**13**^ **C{** ^**1**^ **H} NMR** (101 MHz, CD_2_Cl_2_) *δ* 162.3 (q, ^1^ *J*_CB_ = 50, Ar^F^), 155.1 (s, py), 140.5 (br, py), 135.4 (s, Ar^F^), 129.4 (qq, ^2^ *J*_FC_ = 32, ^3^ *J*_CB_ = 3, Ar^F^), 125.3 (q, ^1^ *J*_FC_ = 273, Ar^F^), 125.0 (br, py), 124.1 (br, imid), 120.2 (br, imid), 118.0 (pent., ^3^ *J*_FC_ = 4, Ar^F^), 57.1 (s, pyCH_2_), 54 (obscured, *N*CH_2_), 31.4 (s, CH_2_), 27.5 (br, CH_2_), 25.8 (s, CH_2_). The carbene resonance was not unambiguously identified in the ^13^C{^1^H} NMR spectrum, but can be located at *ca. δ* 181 from an HMBC experiment. **ESI-MS** (CH_3_CN, 180 °C, 3 kV) positive ion: 512.1927 *m*/*z*, [M]^+^ (calc. 512.1938). **Anal.** Calcd for C_57_H_47_AgBF_24_N_5_·CHCl_3_ (1496.05 g mol^–1^): C, 46.28; H, 3.22; N, 4.64. Found: C, 46.08; H, 3.23; N, 4.58.

### Synthesis of **3**

A suspension of **1**·2HBr (200 mg, 0.353 mmol) and CuBr (152 mg, 1.057 mmol) in THF (7 mL) under argon was cooled to –78 °C before addition of a solution of KO^*t*^Bu (100 mg, 0.881 mmol) in THF (2 mL) *via* cannula. The resulting orange suspension was warmed to room temperature and sonicated to produce a yellow suspension, which was stirred for a further 30 h. The reaction mixture was filtered and the product precipitated by addition of excess pentane, isolated by filtration, washed with pentane and dried. Yield: 147 mg (76%, yellow powder). Crystals suitable for X-ray diffraction were grown from CD_2_Cl_2_/pentane at 20 °C.

^**1**^ **H NMR** (400 MHz, CD_2_Cl_2_) *δ* 7.78 (t, ^3^ *J*_HH_ = 7.7, 1H, py), 7.39 (d, ^3^ *J*_HH_ = 7.7, 2H, py), 7.22 (d, ^3^ *J*_HH_ = 1.7, 2H, imid), 6.98 (d, ^3^ *J*_HH_ = 1.7, 2H, imid), 5.37 (s, 4H, pyCH_2_), 4.17 (t, ^3^ *J*_HH_ = 6.8, 4H, *N*CH_2_), 1.83 (app. pent., *J* = 7, 4H, CH_2_), 1.14–1.34 (m, 16H, CH_2_). ^**13**^ **C{** ^**1**^ **H} NMR** (101 MHz, CD_2_Cl_2_) *δ* 178.7 (s, NCN), 155.5 (s, py), 139.4 (s, py), 123.2 (s, py), 122.5 (s, imid), 120.8 (s, imid), 56.3 (s, pyCH_2_), 52.1 (s, *N*CH_2_), 31.2 (s, CH_2_), 28.1 (s, CH_2_), 27.8 (s, CH_2_), 25.8 (s, CH_2_). **ESI-MS** (CH_3_CN, 180 °C, 3 kV) positive ion: 468.2185 *m*/*z*, [M]^+^ (Calc. 468.2183). **Anal.** Calcd for C_50_H_70_Br_4_Cu_4_N_10_ (1384.96 g mol^–1^): C, 43.36; H, 5.09; N, 10.11. Found: C, 43.28; H, 4.97; N, 9.99.

### Synthesis of **4**

A suspension of **3** (53 mg, 0.0383 mmol) and Na[BAr^F^_4_] (90 mg, 0.1016 mmol) was sonicated in toluene (3 mL) and then stirred at room temperature for 2 days before filtration. The filtrate was concentrated to dryness and the crude product crystallised from THF/pentane. Yield: 62 mg (61%, pale yellow powder) as **4**·0.5THF. Crystals suitable for X-ray diffraction were grown from ether/pentane at 20 °C.

^**1**^ **H NMR** (400 MHz, CD_2_Cl_2_) *δ* 7.83 (t, ^3^ *J*_HH_ = 7.8, 1H, py), 7.73 (bs, 8H, Ar^F^), 7.56 (br, 4H, Ar^F^), 7.42 (d, ^3^ *J*_HH_ = 7.8, 2H, py), 7.10 (d, ^3^ *J*_HH_ = 1.2, 2H, imid), 7.02 (d, ^3^ *J*_HH_ = 1.2, 2H, imid), 5.19 (s, 4H, pyCH_2_), 4.20 (t, ^3^ *J*_HH_ = 7.6, 4H, *N*CH_2_), 1.87 (app. pent., *J* = 7, 4H, CH_2_), 1.25–1.48 (m, 16 H, CH_2_).^**13**^ **C{** ^**1**^ **H} NMR** (101 MHz, CD_2_Cl_2_) *δ* 180.3 (s, NCN), 162.3 (q, ^1^ *J*_CB_ = 50, Ar^F^), 153.8 (s, py), 140.2 (s, py), 135.4 (s, Ar^F^), 129.5 (qq, ^2^ *J*_FC_ = 32, ^3^ *J*_CB_ = 3, Ar^F^), 125.2 (q, ^1^ *J*_FC_ = 271, Ar^F^), 124.1 (s, py), 123.2 (s, imid), 119.6 (s, imid), 118.1 (pent., ^3^ *J*_FC_ = 4, Ar^F^), 54.9 (s, pyCH_2_), 52.9 (s, *N*CH_2_), 31.4 (s, CH_2_), 27.2 (s, CH_2_), 27.1 (s, CH_2_), 25.8 (s, CH_2_), 25.6 (s, CH_2_). **ESI-MS** (CH_3_CN, 180 °C, 3 kV) positive ion: 468.2185 *m*/*z*, [M]^+^ (Calc. 468.2183). **Anal.** Calcd for C_57_H_47_BCuF_24_N_5_ (1332.33 g mol^–1^): C, 51.38; H, 3.56; N, 5.26. Found: C, 51.48; H, 3.47; N, 5.34.

### *In situ* formation of **5** and **6**

A J. Young's NMR tube was charged with 0.004/0.008 mmol of **2/4** and 1.1 equivalent (metal per metal) of [Pd(NCMe)_2_Cl_2_]/[Rh(CO)_2_Cl]_2_. CD_2_Cl_2_ (0.5 mL) was added and the reaction monitored by ^1^H NMR until complete consumption of **2/4**. The samples were periodically placed in an ultrasound bath during the course of the reaction. In the case of the reaction between **4** and [Rh(CO)_2_Cl]_2_, the crude reaction mixture was subsequently passed through a short silica plug (pipette, 3 cm) with additional CH_2_Cl_2_ to afford **6** in 82% yield following removal of the solvent *in vacuo*.

### Synthesis of **7**

#### Following *in situ* reaction monitoring

A J. Young's NMR tube was charged with 0.004/0.008 mmol of **2/4** and 1.4 equivalent (metal per metal) of [NiCl_2_(gylme)]. CD_2_Cl_2_ (0.5 mL) was added and the reaction monitored by ^1^H NMR at either 20 °C or 40 °C. The samples were periodically placed in an ultrasound bath during the course of the reaction. On complete consumption of **2/4** at 40 °C, the crude reaction mixture was passed through a short alumina plug (pipette, 3 cm) with additional CH_2_Cl_2_ to afford **7** in 27%/26% yield following removal of the solvent *in vacuo*.

#### Using *in situ* silver-based transmetallation reagent

A mixture of **1**·2HBr (100 mg, 0.176 mmol), Ag_2_O (45 mg, 0.194 mmol) and Na[BAr^F^_4_] (172 mg, 0.194 mmol) was suspended in CH_2_Cl_2_ (3 mL) and stirred under nitrogen in the absence of light for 20 hours. The resulting grey suspension was allowed to settle and the solution filtered into a flask charged with solid [NiCl_2_(glyme)] (40 mg, 0.182 mmol). After stirring the resulting suspension for 6 h, the solution was passed through a short alumina plug (pipette, 3 cm, washed with CH_2_Cl_2_) and the product obtained on removal of the volatiles *in vacuo*. Yield = 70 mg (29%, yellow powder). Crystals suitable for X-ray diffraction were grown from a mixture of toluene, diethylether, cyclohexane and pentane at 20 °C.

^**1**^ **H NMR** (500 MHz, CD_2_Cl_2_): *δ* 7.82 (t, ^3^ *J*_HH_ = 7.7, 1H, py), 7.68–7.76 (m, 8H, Ar^F^), 7.55 (br, 4H, Ar^F^), 7.45 (d, ^3^ *J*_HH_ = 7.7, 2H, py), 7.11 (d, ^3^ *J*_HH_ = 1.7, 2H, imid), 6.87 (d, ^3^ *J*_HH_ = 1.7, 2H, imid), 6.30 (d, ^2^ *J*_HH_ = 15.0, 2H, pyCH_2_), 5.14 (d, ^2^ *J*_HH_ = 15.0, 2H, pyCH_2_), 4.73 (app. t, *J* = 12, 2H, *N*CH_2_), 3.68–3.78 (m, 2H, *N*CH_2_), 1.94 (br, 2H, CH_2_), 1.66 (br, 2H, CH_2_), 1.18–1.50 (m, 14H, CH_2_), 1.09 (br, 2H, CH_2_). ^**13**^ **C{** ^**1**^ **H} NMR** (126 MHz, CD_2_Cl_2_): *δ* 162.3 (q, ^1^ *J*_CB_ = 50, Ar^F^), 162.0 (s, NCN), 156.5 (s, py), 140.9 (s, py), 135.3 (s, Ar^F^), 129.4 (qq, ^2^ *J*_FC_ = 32, ^3^ *J*_CB_ = 3, Ar^F^), 125.2 (q, ^1^ *J*_FC_ = 271, Ar^F^), 125.1 (s, py), 123.0 (s, imid), 121.4 (s, imid), 118.0 (pent., ^3^ *J*_FC_ = 4, Ar^F^), 55.0 (s, pyCH_2_), 51.3 (s, *N*CH_2_), 30.8 (s, CH_2_), 28.7 (s, CH_2_), 27.5 (s, CH_2_), 23.7 (s, CH_2_). **ESI-MS** (CH_3_CN, 180 °C, 4 kV) positive ion: 498.1929 *m*/*z*, [M]^+^ (calc. 498.1929). **Anal.** Calcd for C_57_H_47_BClF_24_N_5_Ni (1362.95 g mol^–1^): C, 50.23; H, 3.48; N, 5.14. Found: C, 50.62; H, 3.74; N, 5.05.

### Crystallography

Full details about the collection, solution and refinement are documented in the CIF, which have been deposited with the Cambridge Crystallographic Data Centre under CCDC 1470494–1470497.
