# Supplementary material for: Well-defined coinage metal transfer agents for the synthesis of NHC-based nickel, rhodium and palladium macrocycles
Source: Dalton Trans. 2016 May 9;45(21):8937–44. doi: 10.1039/c6dt01263a (PMC5783018; doi:10.1039/c6dt01263a)
Supplement: Supplementary file 1 [file DT-045-C6DT01263A-s001.pdf]

Electronic Supplementary Material (ESI) for Dalton Transactions.
This journal is © The Royal Society of Chemistry 2016

Supporting material:

Well-defined coinage metal transfer agents for the synthesis of NHC-based nickel, rhodium and palladium macrocycles

Rhiann E. Andrew, Caroline M. Storey and Adrian B. Chaplin*

Department of Chemistry, University of Warwick, Gibbet Hill Road, Coventry CV4 7AL, UK.

E-mail: a.b.chaplin@warwick.ac.uk

Contents:

1. **Selected NMR spectra of 2, 3, 4 and 7** S2
   1.1. $[Ag(\mathbf{1})]_2[BAr^F_4]_2$ (**2**) S2
   1.2. $[Cu(\mathbf{1})]_2[Cu_2Br_4]$ (**3**) S4
   1.3. $[Cu(\mathbf{1})][BAr^F_4]$ (**4**) S5
   1.4. $[Ni(\mathbf{1})][BAr^F_4]$ (**7**) S6
2. **NMR-scale Transmetallation Reactions of 2 and 4** S7
   2.1. Reaction of **2** with $[PdCl_2(NCMe)_2]$ to give **5** S7
   2.2. Reaction of **4** with $[PdCl_2(NCMe)_2]$ to give **5** S7
   2.3. Reaction of **2** with $[Rh(CO)_2Cl]_2$ to give **6** S8
   2.4. Reaction of **4** with $[Rh(CO)_2Cl]_2$ to give **6** S8
   2.5. Reactions of **2** with $[NiCl_2(glyme)]$ to give **7** S9
   2.6. Reactions of **4** with $[NiCl_2(glyme)]$ to give **7** S10
3. **High Resolution Mass Spectra of 2, 3, 4 and 7** S11

1. Selected NMR spectra of 2, 3, 4 and 7

1.1. $[Ag(\mathbf{1})]_2[BAr^F_4]_2$ (**2**)

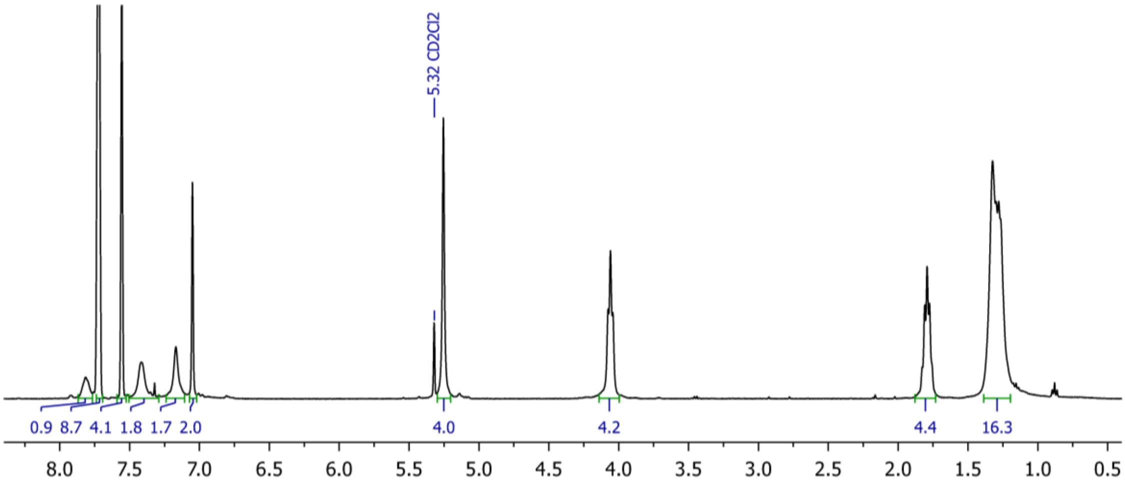

Figure S1: ^{1}H NMR spectrum of **2** (CD_2Cl_2, 400 MHz, 298 K).

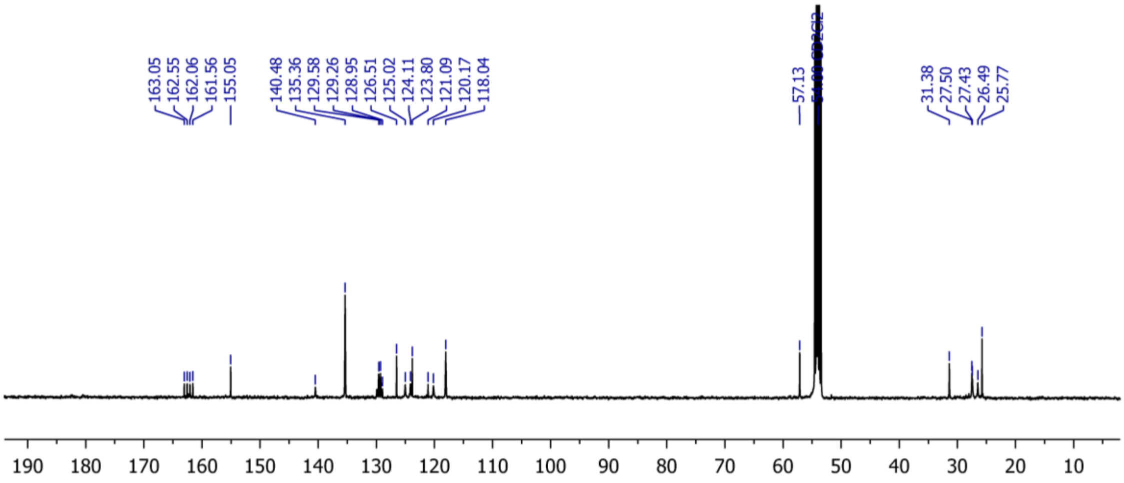

Figure S2: $^{13}C\{^1H\}$ NMR spectrum of **2** (CD_2Cl_2, 101 MHz, 298 K).

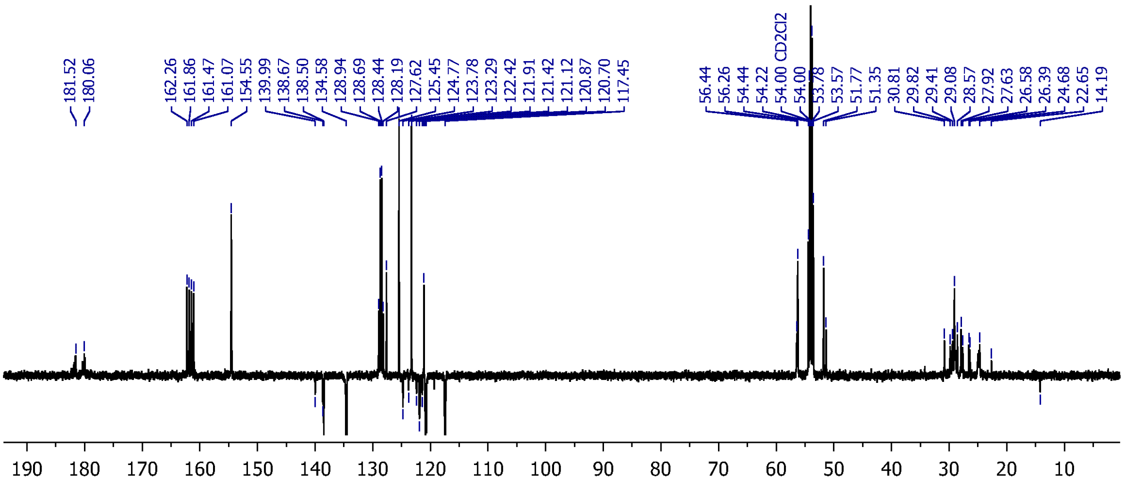

Figure S3: Low temperature $^{13}C\{^1H\}$ APT NMR spectrum of **2** (CD_2Cl_2, 126 MHz, 200 K).

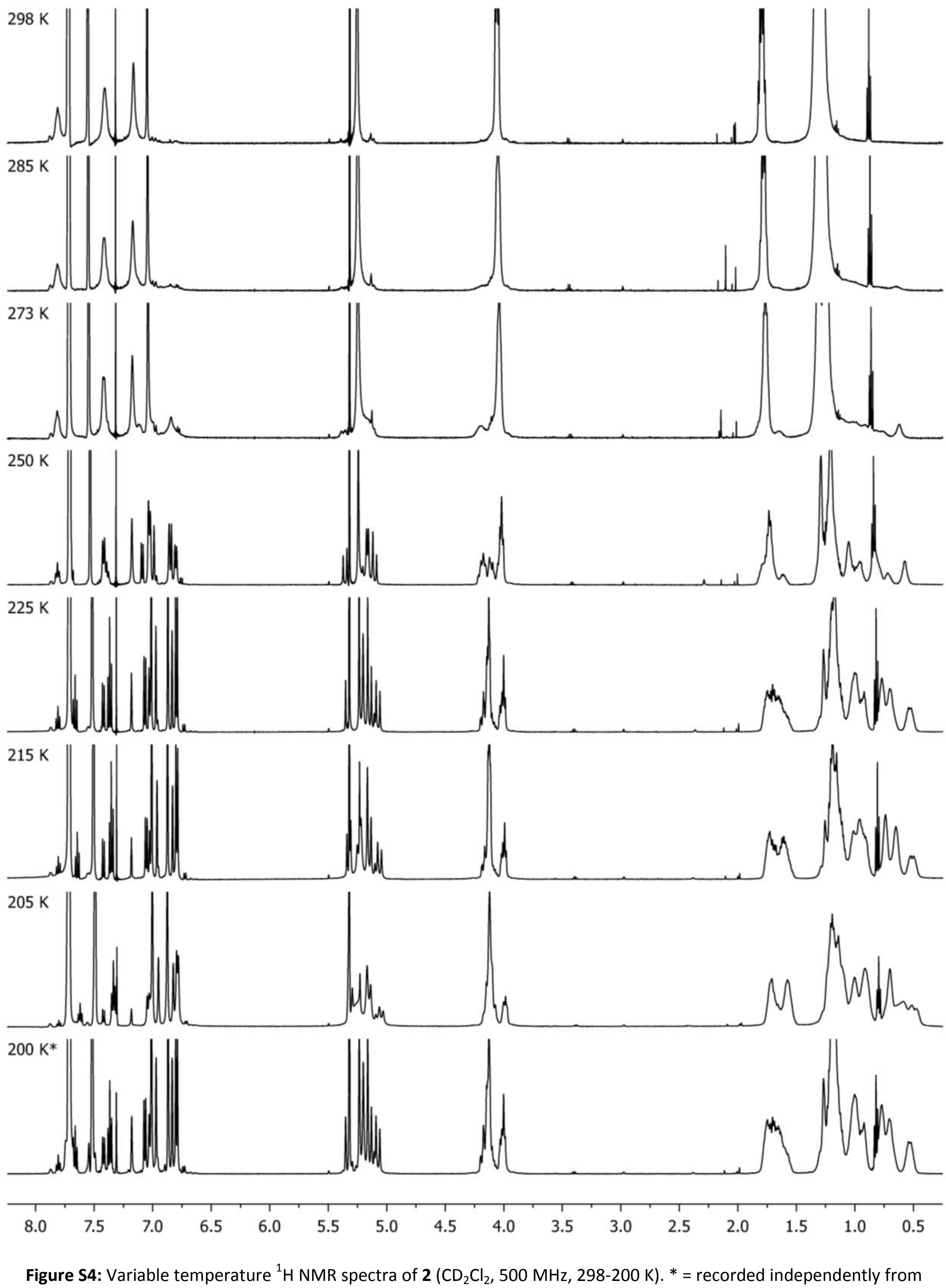

Figure S4: Variable temperature ^{1}H NMR spectra of **2** (CD_2Cl_2, 500 MHz, 298-200 K). * = recorded independently from others

1.2. $[Cu(\mathbf{1})]_2[Cu_2Br_4]$ (**3**)

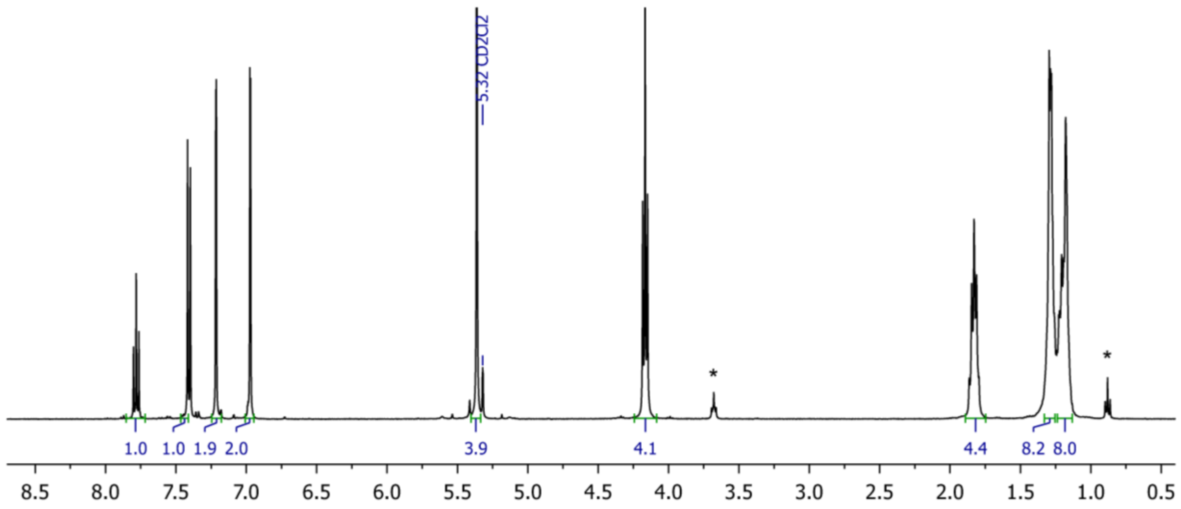

Figure S5: ^{1}H NMR spectrum of **3** (CD_2Cl_2, 400 MHz). * = THF, pentane.

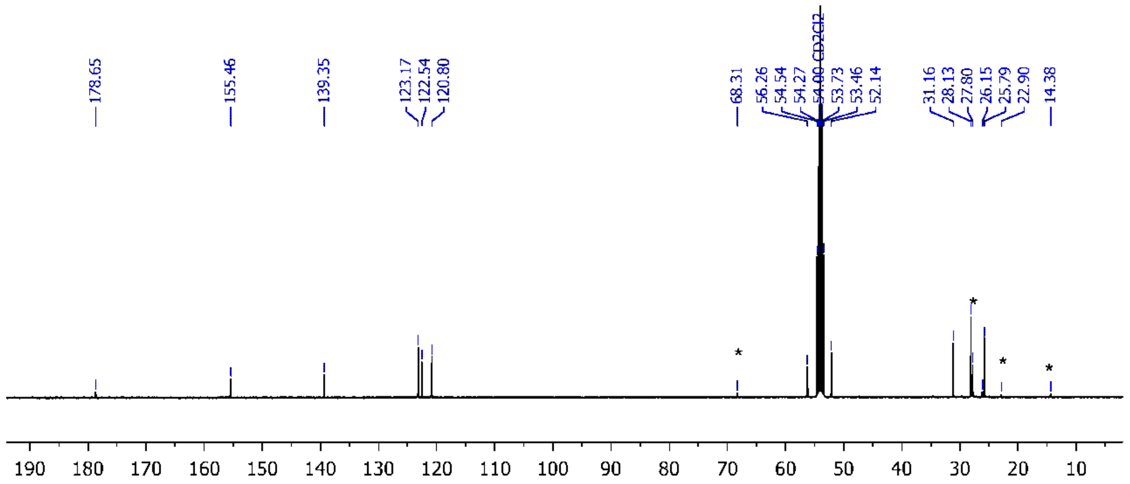

Figure S6: $^{13}C\{^{1}H\}$ NMR spectrum of **3** (CD_2Cl_2, 101 MHz). * = THF, pentane.

1.3. [Cu(**1**)][BAr$^F_4$] (**4**)

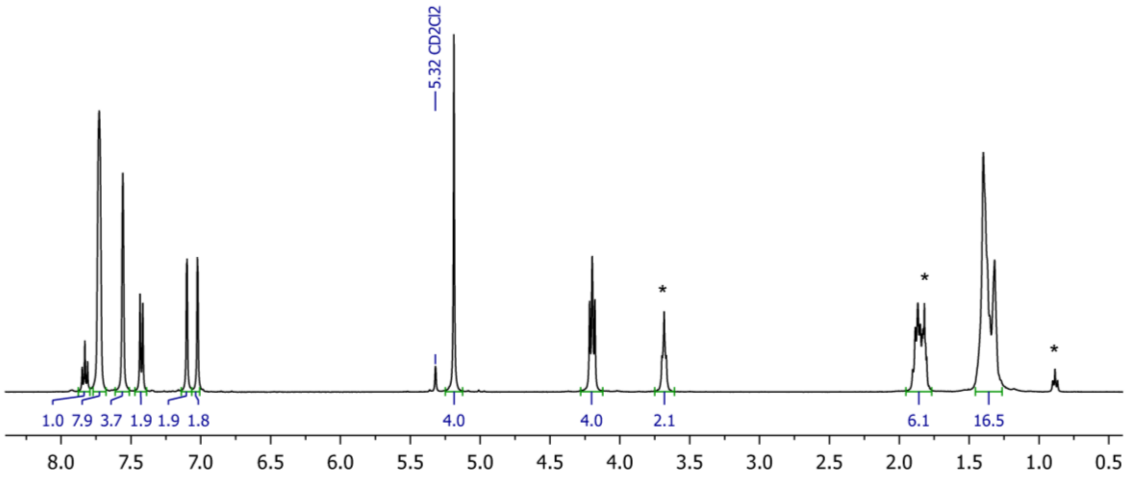

Figure S7: ^{1}H NMR spectrum of **4** (CD_2Cl_2, 400 MHz). * = THF, pentane.

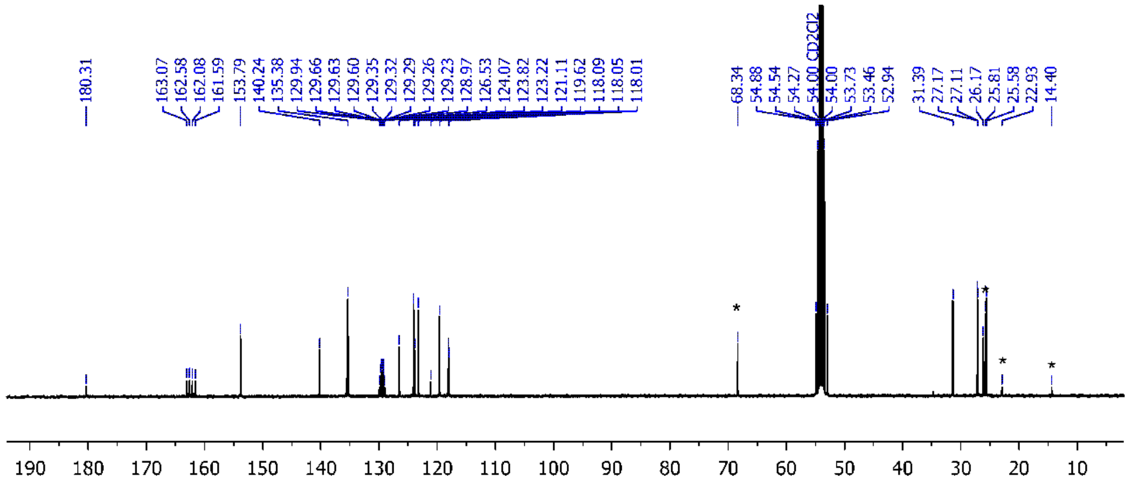

Figure S8: $^{13}C\{^1H\}$ NMR spectrum of **4** (CD_2Cl_2, 101 MHz). * = THF, pentane.

1.4. [Ni(**1**)][BAr$^F_4$] (**7**)

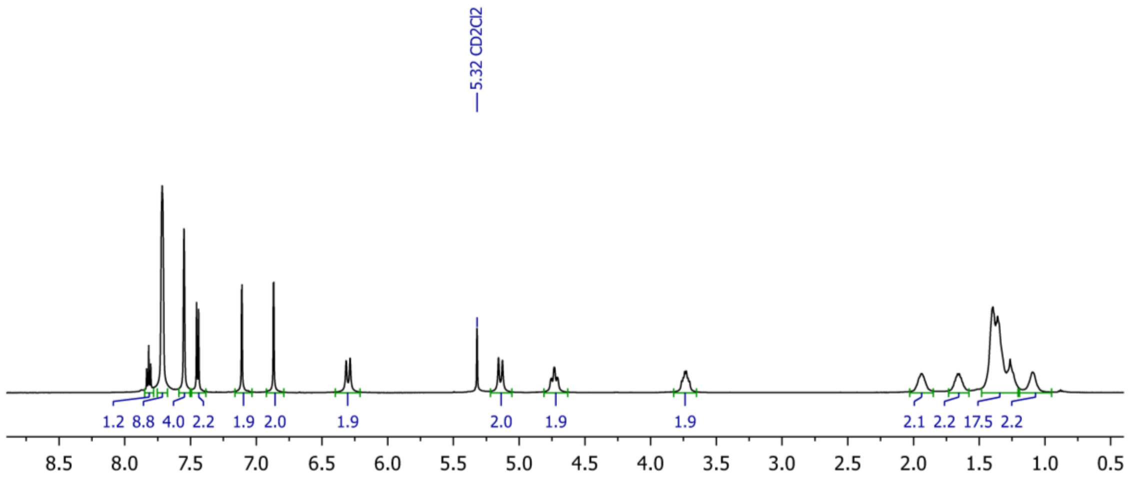

Figure S9: ^1H NMR spectrum of **7** (CD_2Cl_2, 500 MHz).

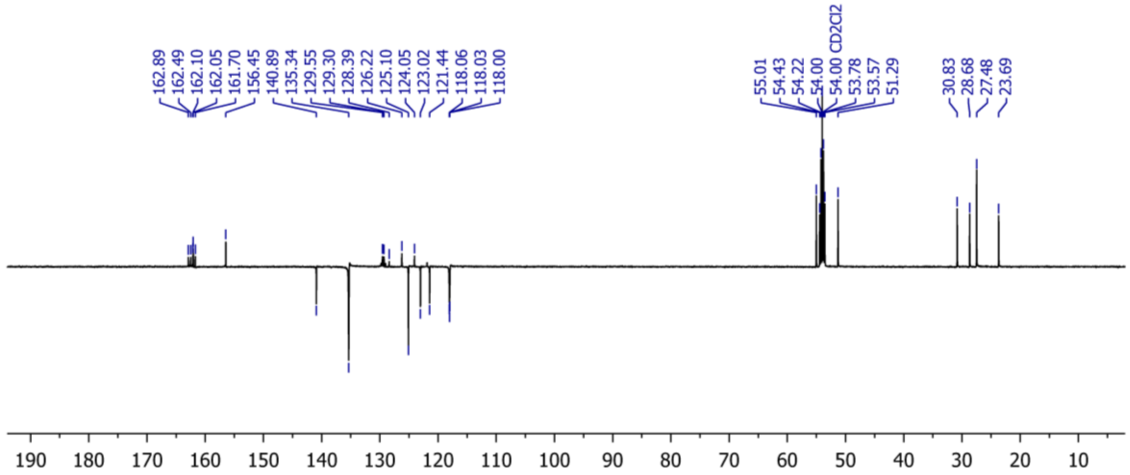

Figure S10: $^{13}C\{^1H\}$ APT NMR spectrum of **7** (CD_2Cl_2, 126 MHz).

2. NMR-scale Transmetallation Reactions of 2 and 4

2.1. Reaction of **2** with $[PdCl_2(NCMe)_2]$ to give **5**

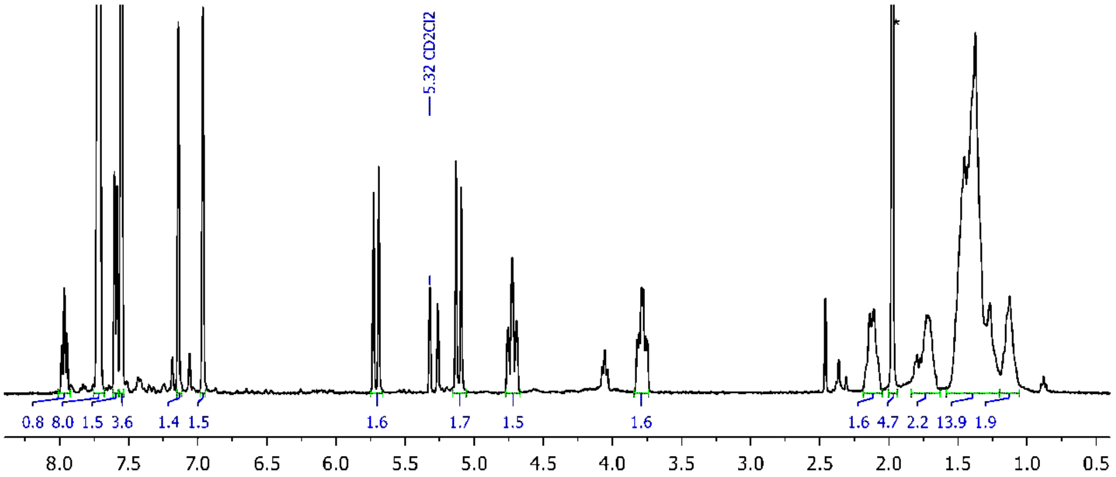

Figure S11: ^{1}H NMR spectrum of *in situ* reaction of **2** and $[PdCl_2(NCMe)_2]$ at 20°C after 0.5 h (CD_2Cl_2, 400 MHz). * = MeCN.

2.2. Reaction of **4** with $[PdCl_2(NCMe)_2]$ to give **5**

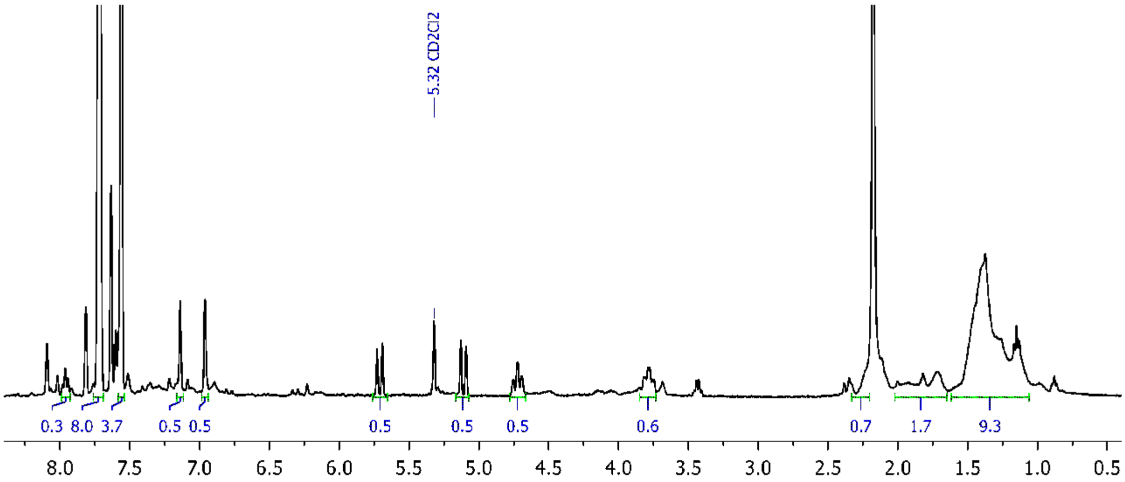

Figure S12: ^{1}H NMR spectrum of *in situ* reaction of **4** and $[PdCl_2(MeCN)_2]$ at 20°C after 0.5 h (CD_2Cl_2, 400 MHz). * = MeCN.

2.3. Reaction of **2** with $[Rh(CO)_2Cl]_2$ to give **6**

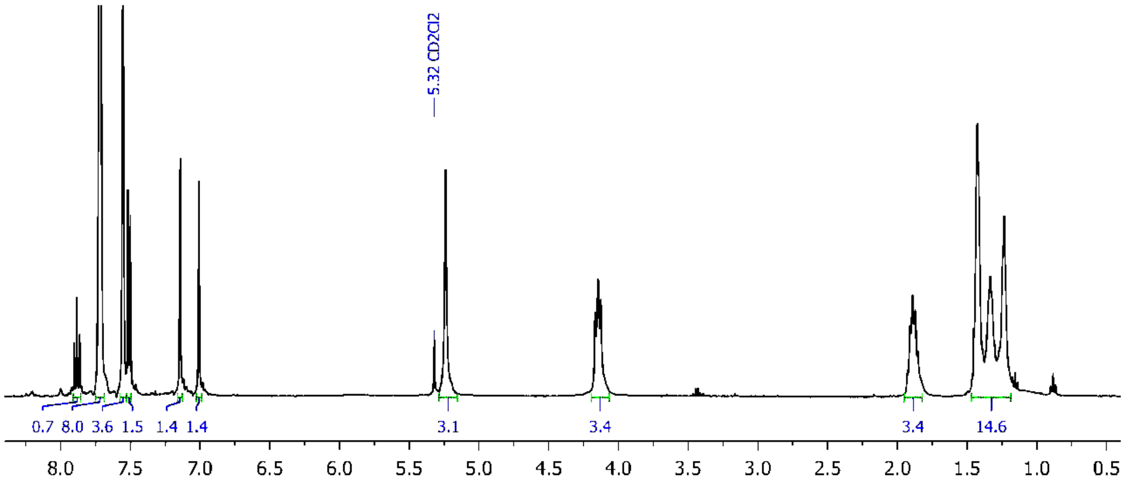

Figure S13: ^{1}H NMR spectrum of *in situ* reaction of **2** and $[Rh(CO)_2Cl]_2$ at 20°C after 0.5 h (CD_2Cl_2, 400 MHz).

2.4. Reaction of **4** with $[Rh(CO)_2Cl]_2$ to give **6**

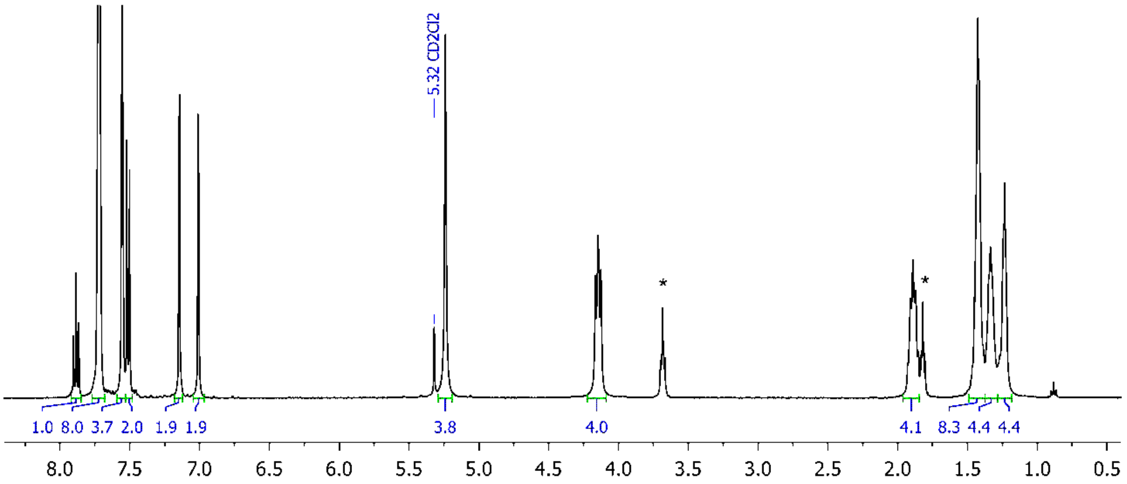

Figure S14: ^{1}H NMR spectrum of *in situ* reaction of **4** and $[Rh(CO)_2Cl]_2$ at 20°C after 0.5 h (CD_2Cl_2, 400 MHz). * = THF

2.5. Reactions of **2** with [$NiCl_2$(glyme)] to give **7**

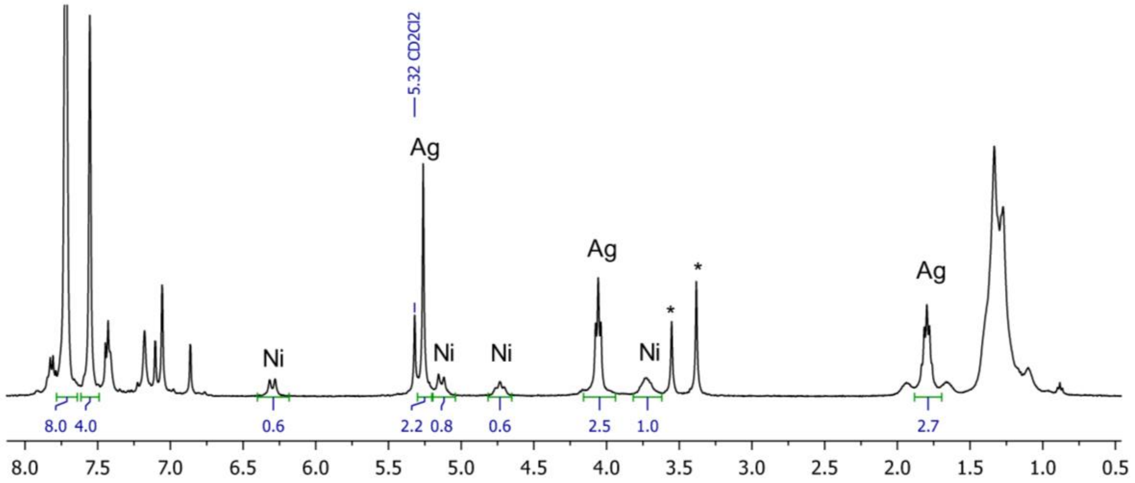

Figure S15: ^{1}H NMR spectrum of *in situ* reaction of **2** and [$NiCl_2$(glyme)] at 20°C after 20 h (CD_2Cl_2, 400 MHz). * = glyme.

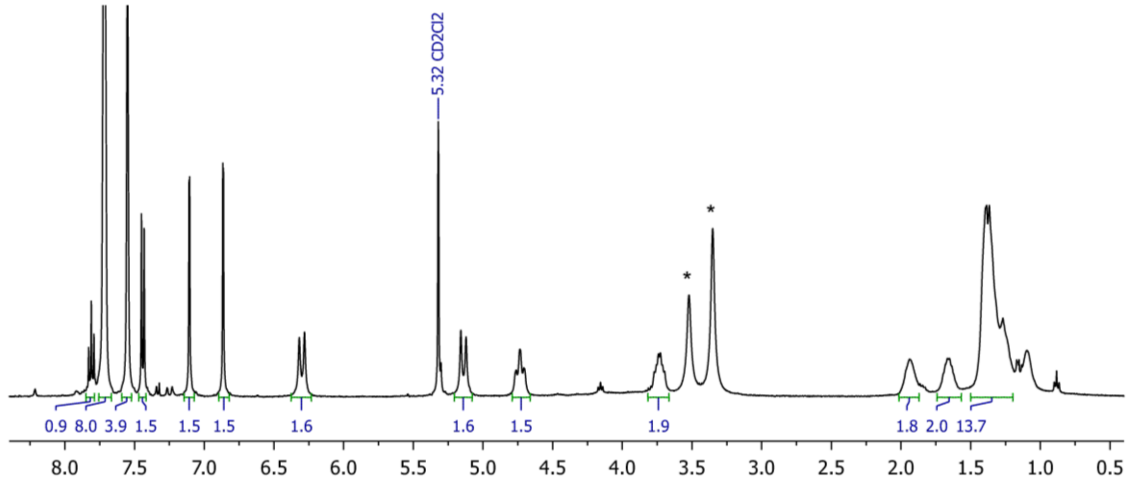

Figure S16: ^{1}H NMR spectrum of *in situ* reaction of **2** and [$NiCl_2$(glyme)] at 40°C after 20 h (CD_2Cl_2, 400 MHz). * = glyme.

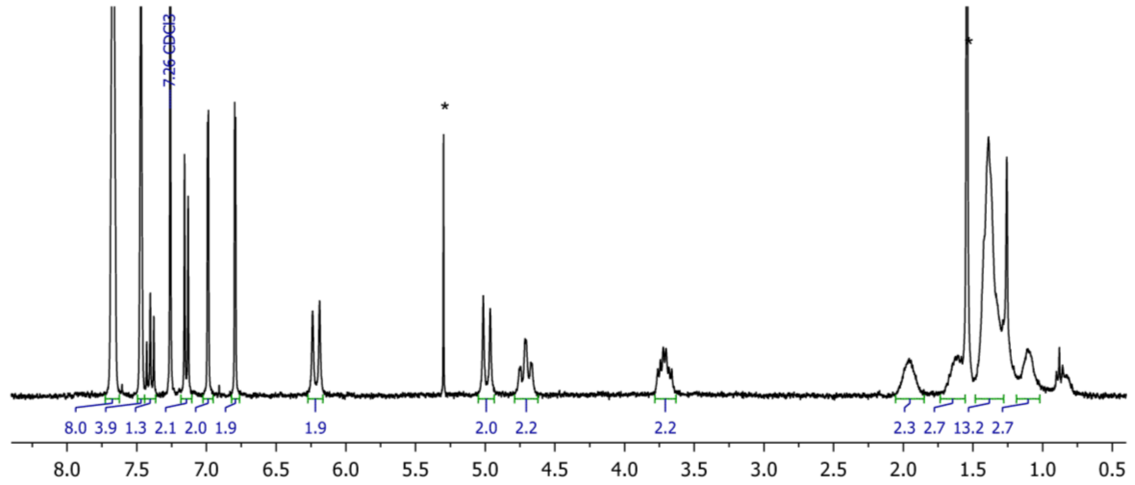

Figure S17: ^{1}H NMR spectrum of isolated **7** from **2** ($CDCl_3$, 300 MHz). * = CH_2Cl_2, H_2O

2.6. Reactions of **4** with [NiCl$_2$(glyme)] to give **7**

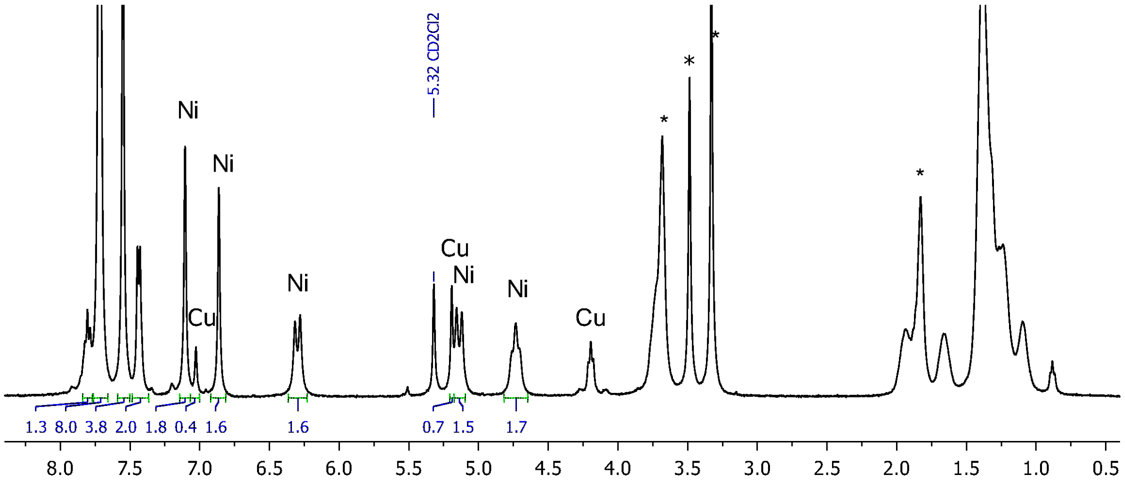

Figure S18: ^{1}H NMR spectrum of *in situ* reaction of **4** and [NiCl$_2$(glyme)] at 20°C after 20 h (CD_2Cl_2, 400 MHz). * = THF, glyme.

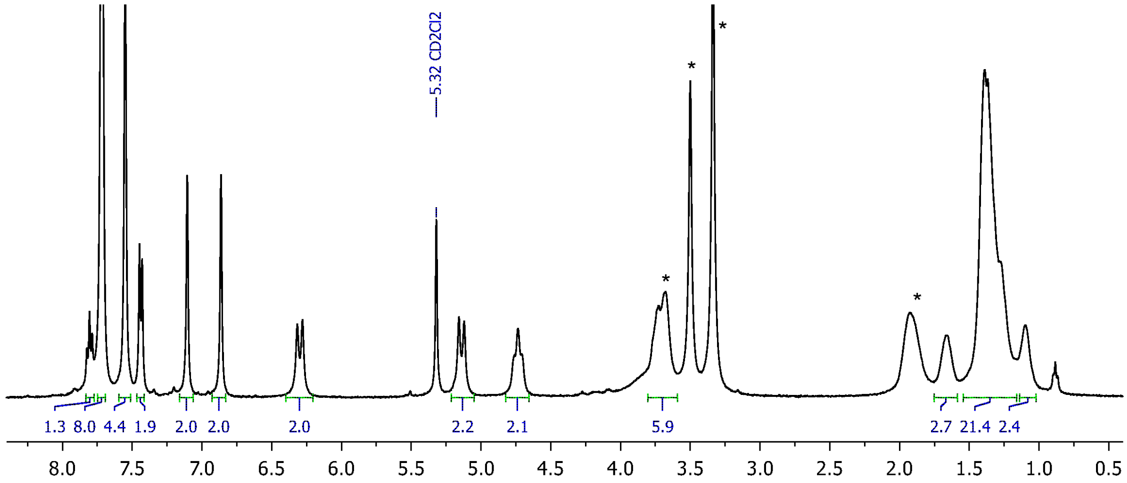

Figure S19: ^{1}H NMR spectrum of *in situ* reaction of **4** and [NiCl$_2$(glyme)] at 40°C after 20 h (CD_2Cl_2, 400 MHz). * = THF, glyme.

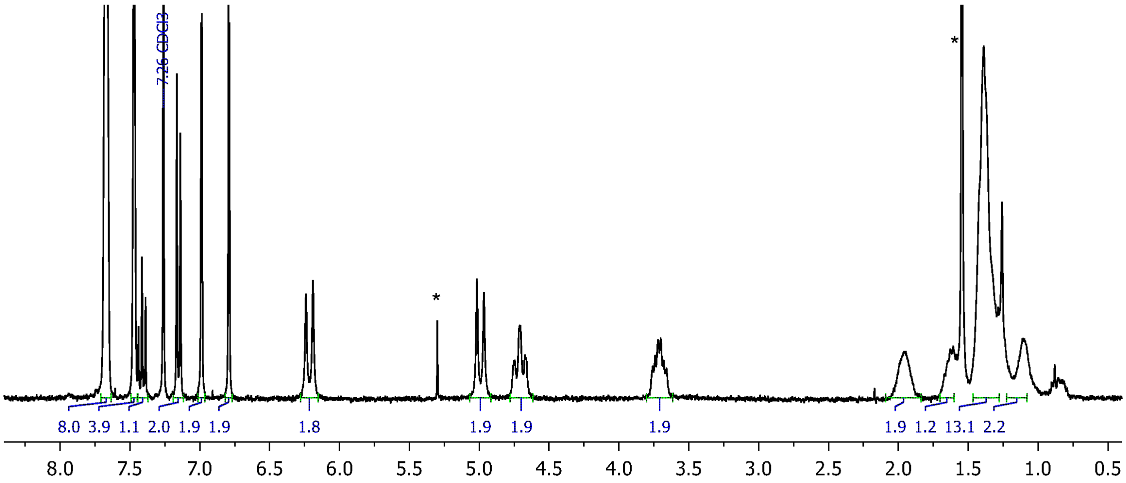

Figure S20: ^{1}H NMR spectrum of isolated **7** from **4** ($CDCl_3$, 300 MHz). * = CH_2Cl_2, H_2O

3. High Resolution Mass Spectra of 2, 3, 4 and 7

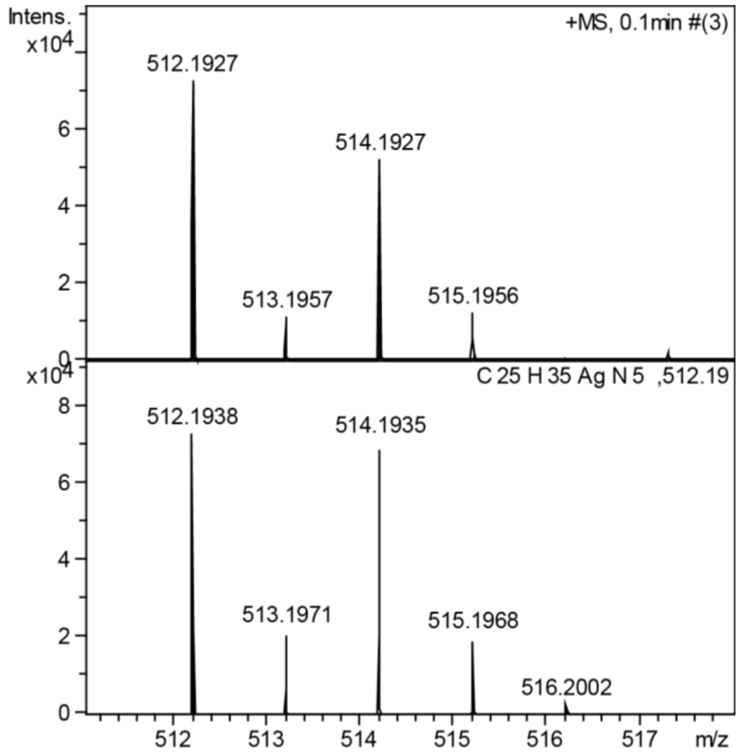

Figure S21: ESI-MS spectrum of **2**.

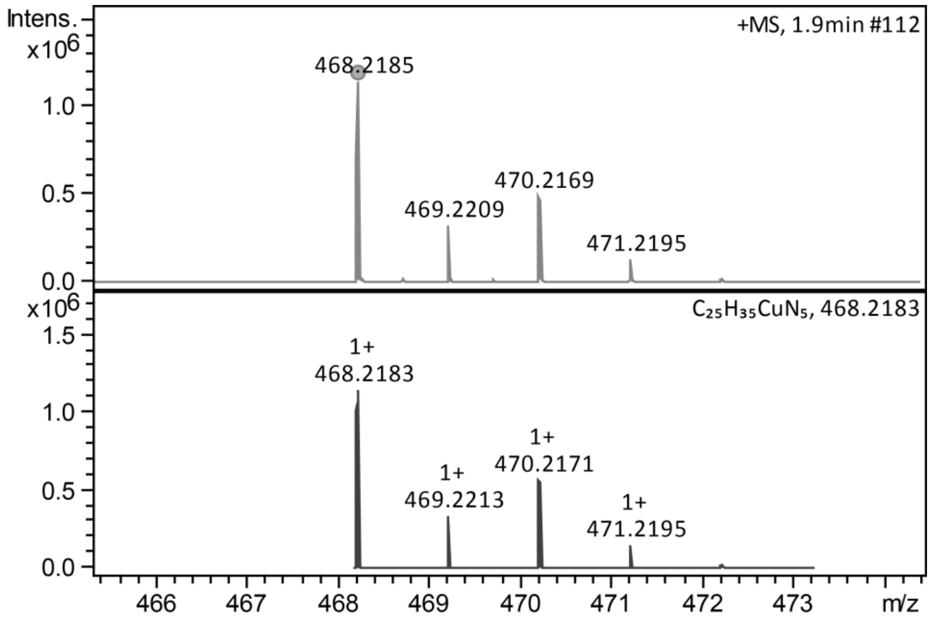

Figure S22: ESI-MS spectrum of **3**.

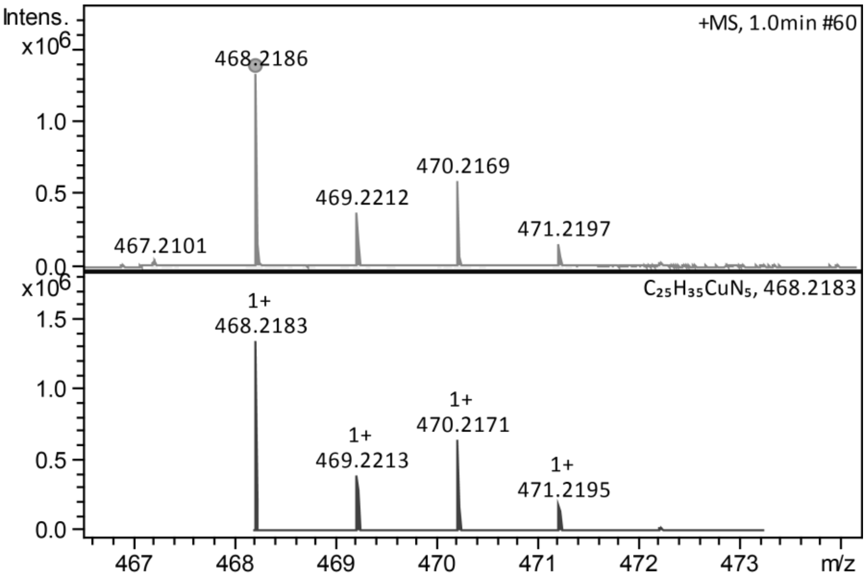

Figure S23: ESI-MS spectrum of **4**.

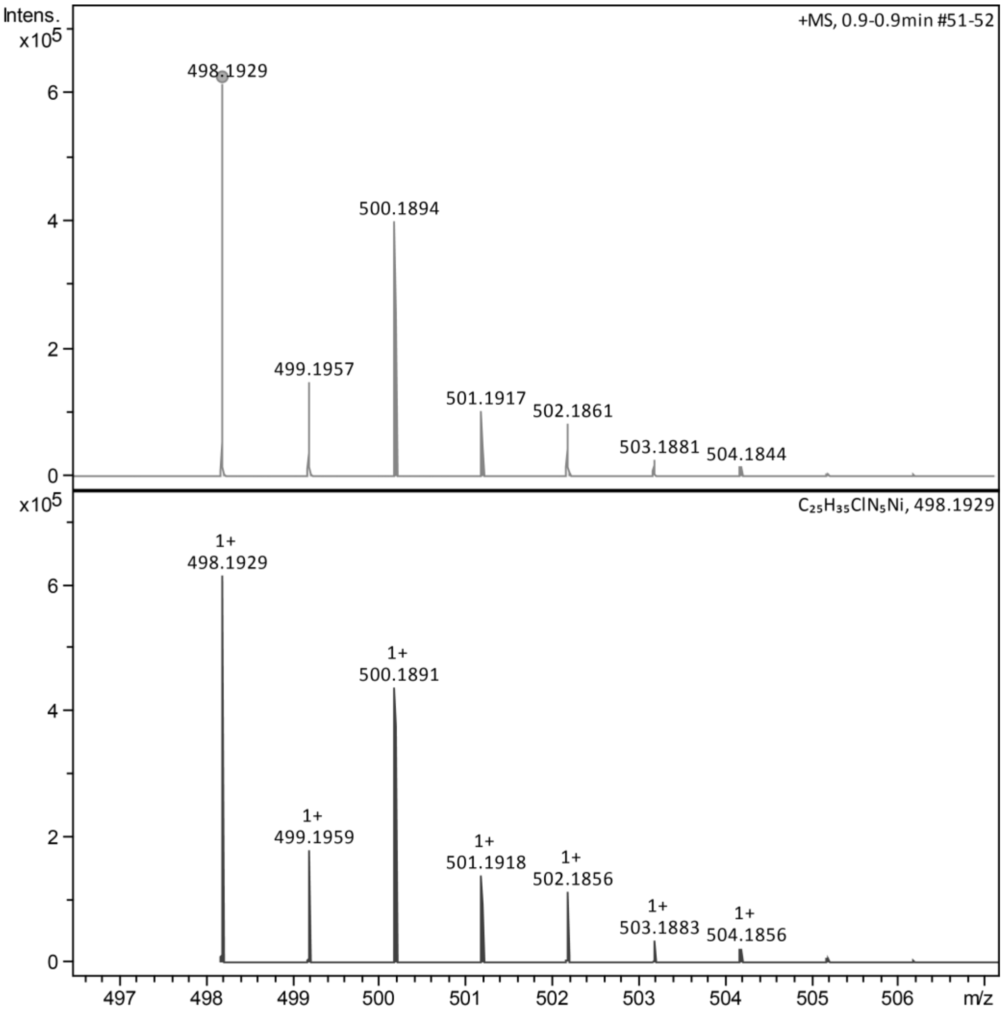

Figure S24: ESI-MS spectrum of **7**.
